# Mapping the Path to Cognitive Balance: Applying the States of Mind Model and Network Analysis to Eating Disorder Patients

**DOI:** 10.3390/jcm12185790

**Published:** 2023-09-06

**Authors:** Lucia Tecuta, Giuliano Tomei, Raymond DiGiuseppe, Romana Schumann, Donatella Ballardini, Elena Tomba

**Affiliations:** 1 Department of Psychology, University of Bologna, 40127 Bologna, Italy; lucia.tecuta2@unibo.it (L.T.); giuliano.tomei2@unibo.it (G.T.); 2 Department of Psychology, St. John’s University, New York, NY 11439, USA; digiuser@stjohns.edu; 3 Eating Disorder Clinic “Centro Gruber”, 40125 Bologna, Italy; r.schumann.gruber@gmail.com (R.S.); donatella.ballardini@yahoo.com (D.B.)

**Keywords:** cognitions, irrational beliefs, rational beliefs, eating disorders, psychological well-being, network analysis

## Abstract

**Background**: In eating disorders (EDs), cognitive-behavioral therapy (CBT) represents one of the first-line treatment options albeit with sub-optimal results. The assessment of cognitive balance through an index measuring increased adaptive thinking and reduced maladaptive thinking, the desired outcomes, and the ultimate goal of CBT treatments warrants attention. The states of mind model (SOM) provides a framework through which a cognitive balance index can be defined. The current cross-sectional controlled study tested the clinical utility of the SOM model in a sample of ED outpatients. **Methods**: ED outpatients (n = 199) were assessed at baseline with the attitudes and beliefs scale-2 (ABS-2) for rational beliefs (RBs) and irrational beliefs (IBs), from which a SOM ratio score index (RBs/(RBs + IBs)) was calculated, the eating disorder inventory-3 (EDI-3) for ED symptoms and ED-related psychopathological features, the psychological well-being scales (PWB) for positive psychological functioning. A matched control sample (n = 95) was also assessed with the ABS-2. **Results**: ED patients exhibited significantly lower SOM and RB scores compared to controls. Network analysis results highlighted the centrality of the SOM-cognitive balance index, PWB-self-acceptance, and EDI-3-general psychological maladjustment, as well as the importance of the influence that cognitive balance and general psychological maladjustment exert on each other. **Conclusions**: The findings support the clinical utility of the SOM ratio applied to cognitions in EDs. This demonstrates its ability to differentiate such patients from controls and in capturing worse ED-related general psychopathology as well as compromised aspects of psychological well-being, in particular self-acceptance and environmental mastery. It thus might be considered in CBT treatment of EDs a potential cognitive clinimetric and clinical index of ED severity indicating key difficulties in counteracting maladaptive thinking with adaptive thinking.

## 1. Introduction

Cognitively oriented therapies, whose main targets are dysfunctional cognitions, are considered first-line treatments for eating disorders (EDs) [1,2]. Females are dispropor-tionately affected by EDs and, compared with male counterparts, report greater severity of ED symptoms [3,4]. Indeed, females generally report greater cognitive vulnerability in terms of early maladaptive schemas compared with males [5,6] and in association with sexual and marital dissatisfaction [7,8] as well as in association with eating attitudes [1]. While the empirical relationship between maladaptive cognitions such as negative self-beliefs and ED symptoms has been extensively supported in females with EDs [9,10,11,12,13,14,15,16,17] the assessment of functional adaptive beliefs regarding the self, others, and the world in this clinical population is frequently ignored [14,18].

Growing research in this field is clinically relevant. Firstly, a healthy balance of positive and negative thinking is the ultimate therapeutic goal of traditional cognitive behavioral therapy (CBT) models, which define the formulation of alternative functional thoughts and belief revision as necessary for lasting cognitive change [19,20]. Second, in a metanalysis by Tomba et al. [21], cognitive functioning was found to remain significantly compromised in EDs even after engaging in effective treatment, perhaps representing enduring residual symptomology and an important target of focused clinical attention. Indications on how to comprehensively assess cognitive profiles in EDs are currently lacking in clinical guidelines [1,22,23]. Not much is known about how balanced thinking styles may be associated with patients’ psychological symptoms in terms of both ED symptoms and ED-related psychopathological features.

Furthermore, not much is known about how thinking styles in terms of balanced thinking may be associated with patients’ functional outcomes such as psychological well-being. The evaluation of positive psychological functioning constitutes an additional fundamental area in ED research which, albeit being frequently neglected, has been shown to play an important role in ED symptomatology and remission in a few studies [24,25] as well as being considered a factor of increased vulnerability and adversity in other psychiatric populations [26,27].

A theoretical model, the states of mind (SOM) model, offers an index of balanced thinking, named “cognitive balance”, useful for research and clinical settings [28,29]. It has found support both in general [30,31,32,33,34,35] and clinical populations, including social phobia [36] and agoraphobia [37] and post-traumatic stress disorder [38]. The SOM considers the presence of both functional and dysfunctional cognitions simultaneously as fundamental elements of the way individuals organize and appropriately process cognitive information. The SOM theorizes that individuals apply an internal computation system in balancing their cognitions asymmetrically (positive cognitions/(positive + negative cognitions)). Specifically, psychopathology would emerge when a persistent shift from the optimal cognitive balance occurs and thoughts are mostly negative. Optimal cognitive balance instead would be characterized by a predominance of positive cognitions over negative ones. The SOM model offers a rare definition of cognitive processes and mechanisms that can be empirically studied but also routinely applied in clinical psychotherapeutic contexts.

The current study’s aim was to investigate a SOM-derived index of cognitive balance in EDs and controls and its associations with ED-related symptomatology, general psychological maladjustment, and psychological well-being. Moreover, the present study aimed to examine in ED outpatients the relationship between SOM cognitive balance and both ED psychopathology (ED symptoms and ED-related psychopathological features) and psychological well-being through psychometric network analysis (a novel statistical methodology that offers alternative ways of analyzing data and of modeling and simulating psychopathological processes) [39].

The first hypothesis concerned ED outpatients exhibiting significantly lower SOM cognitive balance, that is, less positively skewed beliefs and more negatively-skewed beliefs, compared with a general population sample. These findings will support the clinical utility of the SOM ratio in differentiating ED patients from controls and its feasibility as a possible clinical index of compromised cognitive balance during CBT treatment of EDs. Compromised SOM cognitive balance is hypothesized to represent an important and central aspect in the psychological network of ED patients, as well as exhibit close and strong positive associations with ED psychopathology and strong negative associations with dimensions of psychological well-being.

## 2. Materials and Methods

The study was conducted in accordance with the Helsinki Declaration (revised in Fortaleza, Brazil, in October 2013); the relevant sections of the International Conference on Harmonization and Good Clinical Practice (ICH-GCP) (document EMA/CHMP/ICH/135/1995, December 2016) were implemented during the course of the study. Study participation was voluntary and could be canceled at any time without provision of reasons or negative consequences. The project was approved by the Bologna University Bioethics Committee and Department of Psychology Ethics Committee, Bologna University (ethics committee approval no. 68444 on the 10th of May 2018. Informed consent was obtained from all individual participants included in the study prior to data collection.

### 2.1. Participants and Inclusion Criteria

A convenience sample of consecutively screened outpatients who met the Diagnostic and Statistical Manual of Mental Disorders 5 criteria for EDs (DSM-5) [3] (anorexia nervosa (AN), bulimia nervosa (BN), binge-eating disorder (BED), and other specified feeding or eating disorder (OSFED)) were recruited from a specialized outpatient ED treatment clinic before commencing CBT-based treatment integrated with nutritional rehabilitation. The treatment is explained in detail elsewhere [25]. Inclusion criteria were: (a) 18 to 65 years of age, (b) a diagnosis of AN, BN, BED, or OSFED, and (c) within one month of beginning treatment. The exclusion criteria were: (a) lack of capacity to consent for research, (b) ED diagnosis secondary to a physical health or metabolic condition, and (c) comorbid drug/alcohol abuse, psychotic or neurocognitive disorders, acute suicidality, and pregnancy.

Control participants matched for sex and age were recruited online from the adult general population and from regional university campuses with the following inclusion criteria: (a) 18 to 65 years of age and (b) no prior diagnosis of any ED according to DSM-5 diagnostic criteria. Exclusion criteria were: (a) lack of capacity to consent for research and (b) lifetime history of EDs according to DSM-5 diagnostic criteria, either as primary diagnosis or in comorbidity to other mental health and physical conditions.

### 2.2. Procedures

The evaluation of ED outpatients was performed during the first intake visit. ED diagnoses were established at intake by the consensus of a psychiatrist and a clinical psychologist independently using the structured clinical interview for DSM-5 [40]. Each diagnostic interview was conducted and recorded by a clinical psychologist specialized in the assessment of EDs and subsequently reviewed by a consulting psychiatrist also specialized in EDs, who confirmed the diagnosis. PhD-trained clinical psychologists involved in the research (L.T. and E.T.) approached patients in the waiting room with a written presentation of the study. Consent forms and questionnaire packets were left for patients interested in participating and collected by the front desk of the clinic.

Presentation of results and discussion of results follow the Strengthening the Reporting of Observational Studies in Epidemiology (STROBE) guidelines [41].

### 2.3. Instruments

Patients were assessed at baseline before commencing treatment through the following self-rating questionnaires:

The Attitudes and Beliefs Scale 2 (ABS-2) [42,43] was developed within the rational emotive behavioral therapy framework and offers definitions of functional and dysfunctional cognitions in terms of rational beliefs (RBs) and irrational beliefs (IBs) [42]. It is composed of 76 Likert scale items and measures the four irrational and four rational belief processes, respectively, identified by Ellis [44]: demandingness versus non-demanding preferences, awfulizing versus realistic negative expectations, low frustration tolerance versus high frustration tolerance, and negative global evaluation versus self-acceptance. Demands represent rigid, inflexible, and nonpragmatic beliefs and reflect absolutistic “must statements”. Awfulizing statements are instead excessive negative evaluations and expectations of events, while low frustration tolerance beliefs refer to thinking that one cannot tolerate an event or set of circumstances. Negative global self-evaluations/self-downing refer to generalized negative labeling and self-statements. The ABS-2 has demonstrated excellent construct validity pertaining to the four irrational and four rational belief processes [42,43] and good psychometric properties, including good internal consistency and divergent and convergent validity in numerous studies [42,43,45,46]. The Italian translation of the ABS-2 utilized in previous studies was used [47]. This translation has already demonstrated excellent internal consistency in the general Italian college-age population (α = 0.926) [47] as well as in eating disorder samples (α = 0.971) [16], in line with validation studies [42,43]. In the present study, only the total rational beliefs scale and total irrational beliefs scale were used in the calculation of a SOM score (please see Methods section).

The Eating Disorder Inventory 3 (EDI-3) [48] is a self-rating 91-item questionnaire with items constructed on a 5-point 0–4 Likert scale assessing clinically relevant psychological traits and constructs in EDs that has been standardized and translated in numerous languages including Italian. In the current study, the Italian adaptation of the EDI-3 was used [49]. It yields 12 primary scales (3 of which are ED-risk scales and 9 of which are ED-related psychological scales) and the following 6 composite scales: eating disorder risk/severity, ineffectiveness, interpersonal problems, affective problems, overcontrol, and general psychological maladjustment. Only two composite scales were used in the current study. The ED risk scale is composed of 25 items contained in the risk subscales of drive for thinness, bulimia, and body dissatisfaction. The EDI-3 general psychological maladjustment scale is composed of 64 items belonging to all 9 psychological scales: low self-esteem, personal alienation, interpersonal insecurity, interpersonal alienation, interoceptive deficits, emotion dysregulation, perfectionism, asceticism, and maturity fears. This composite score represents a total global psychological functioning index and levels of ED-related psychopathology. The Italian EDI-3 adaptation has shown satisfactory internal consistency (Cronbach’s alpha ranging from 0.70 to 0.94 for subscales in ED patients) and validity. Specifically, for the EDI-3 general psychological maladjustment scale, reported Cronbach alpha was 0.94 [49].

The Psychological Well-being Scale (PWB) [50] is a measure composed of 84 items assessing 6 dimensions of PWB according to Ryff’s model: autonomy, environmental mastery, personal growth, positive relationships with others, purpose in life, and self-acceptance. Items are constructed on a 6-point 1–6 Likert scale, yielding 6 subscale scores ranging from 14 to 84. Cronbach’s alpha coefficients in a sample of 321 individuals from the general population for the 6 scales ranged from 0.86 to 0.93. Test–retest reliability varied between 0.81 and 0.88, whereas validity correlations extended between 0.25 and 0.73 [51]. PWB scales were also administered to controls matched for socio-demographic characteristics. The Italian version was used [52], with the following Cronbach alphas reported by Gremigni and Stuart-Brown [53]: 0.86 for autonomy, 0.78 for environmental mastery, 0.75 for personal growth, 0.84 positive relations, 0.73 for purpose in life, and 0.71 for self-acceptance. All scales were used in the present study.

In ED patients, body mass index (BMI) was collected from updated medical charts that were compiled during the intake process. BMI was calculated by dividing participants’ weight in kilograms by their height in meters squared and was measured using a calibrated scale.

### 2.4. Statistical Analyses

The database used for the analyses presented 0% missing data. The descriptive data (means and standard deviations) were calculated for socio-demographic and clinical characteristics in psychological measures. A Kolmogorov–Smirnov test was performed to evaluate the normality of the descriptive statistics. Independent *t*-tests examined differences in SOM scores between ED outpatients and matched controls.

In the current study, in order to calculate a SOM ratio score, only the ABS-2 total rational (RBs) and ABS-2 total irrational beliefs (IBs) scores were needed. Utilizing ABS-2 scales of rational and irrational beliefs compared with other CBT-related types of cognitions lends greater stability to SOM calculations, as such beliefs (taken from the Rational Emotional Behavior Therapy (REBT) paradigm) represent evaluative aspects of cognitions [54]. As underscored by Cherkasova [32], the SOM scores in previous research have been mainly explored with measures of automatic thoughts or self-statements, which however reflect more surface structure cognitions closely associated with a person’s emotional experience [54]. Following the states of mind model [29], where the proportion of positive (P) to positive plus negative (N) cognitions is represented as P/(P + N) [55], a beliefs proportion score was calculated by replacing P with the ABS-2 RB total score and N with the ABS-2 IB total score. RB/(RB + IB) or SOM score indicates the balance between ABS-2 RBs and ABS-2 IBs, where higher SOM scores indicate higher rationality and thus greater cognitive balance.

Originally, an optimal balance was hypothesized to be represented by the golden-section ratio of 0.62 positive to negative cognitions [29]; however, exact index boundaries for healthy or psychopathological functioning have not been confirmed or supported [32], thus the current study did not examine threshold values.

#### 2.4.1. Network Estimation

Network models were estimated using the *ggmModSelect* estimator for the *estimateNetwork* function from the *bootnet* R package [56]. This algorithm applies regularized estimation to conduct a fast search across model space to recover a network, which is then used as a starting structure in a stepwise model search. Subsequently, the algorithm combines a step-up and step-down approach to test all possible combinations of inclusion and exclusion of each edge of the network. At each iteration, the model with the lowest criterion is selected, until it is no longer possible to improve the information criterion [57]. This technique uses Bayesian information criterion (BIC) obtained through estimating the maximum likelihood of sparsity. Correlations among nodes in *ggmModSelect* networks represent partial correlations in the network while accounting for other symptoms.

#### 2.4.2. Network Accuracy

To assess network accuracy, we applied the *bootstrap* function of the *bootnet* R package [56] to retrieve 2000 bootstrap samples and obtain: (1) a strength centrality stability coefficient (CSC); (2) an edge stability correlation coefficient (ESC). Stability coefficients represent the maximum proportion of cases that can be dropped, such that the correlation between original centrality indices and the reduced sample is at least 0.70. Coefficients between 0.25 and 0.50 are considered acceptable, coefficients above 0.50 and below 0.70 are considered good, and coefficients above 0.70 are considered excellent [56].

#### 2.4.3. Centrality Indices

To identify which nodes were most influential within the network, we opted to estimate the *strength* centrality for every node included in the network. Strength centrality represents the absolute sum of all the weights connected to a node [56]. Nodes with higher strength will tend to be at the center of the network. In psychopathology networks, central symptoms are presumably important for the development and maintenance of the entire network and therefore may represent relevant targets for clinical interventions [39]. In order to avoid the presence of overlapping nodes, which would artificially alter centrality indices [58], controls for topological overlap of nodes were conducted using the *goldbricker* algorithm from the *networktools* R package [59].

## 3. Results

### 3.1. Patient Sample Characteristics

Data from 199 ED outpatients were analyzed (54 with AN, 50 with BN, 60 with OSFED, and 35 with BED) out of 202 invited participants. Only three participants who had been invited to participate refused to fill out questionnaires. Participants were all female, with mean age 27.02 ± 12.44. Mean illness duration was 7.73 ± 9.58 years (range from 6 months to 44 years). Mean BMI at baseline by diagnoses was 16.12 ± 1.55 kg/m^2^ for AN, 22.54 ± 4.767 kg/m^2^ for BN, 34.68 ± 8.61 kg/m^2^ for BED, and 20.37± 3.73 kg/m^2^ for OSFED. Most of the sample were single (n = 170, 85.4%), while the rest were married (n = 25, 12.6%) or separated/divorced (n = 4, 2.0%). Most patients had obtained as their highest degree a high school diploma (n = 100, 50.3%), while 26.6% (n = 53) had graduated from university. The clinical data of the ED sample appear in Table 1.

### 3.2. Control Sample Characteristics

Ninety-five participants from the general population, matched for age and sex, constituted the control sample. The control group was all female, with a mean age of 29 ± 10 years, with a majority of participants being single (n = 79, 83.1%) with either a college (n = 48, 50.5%) or high school (n = 45, 47.3%) degree. Age did not differ significantly from the ED patient sample (*t*(289) = 1.4916, *p* = 0.1369).

### 3.3. Mean Scores of ED Patients in Psychological Measures and T-Test Comparisons in SOM Ratios and Rational Beliefs between ED Patients and Controls

ED patient means and standard deviations in psychological measures including EDI-3 composite scales and PWB scales are shown in Table 1. EDI-3 and PWB scales were not collected from controls, as comparisons in these variables have already been objects of study elsewhere [24,25].

Comparisons between ED outpatients and sex and age-matched controls through independent *t*-tests in SOM ratio scores and rational beliefs scores of the ABS-2 are shown in Table 2, where ED patients exhibited significantly lower ABS-2 SOM ratio scores as well as lower rational beliefs scores with large effect sizes. Irrational beliefs in EDs and comparisons with healthy controls have already been examined in a previous study [16].

### 3.4. Network Analysis with SOM, ED Symptomatology, and Psychological Well-Being

**Figure 1:**
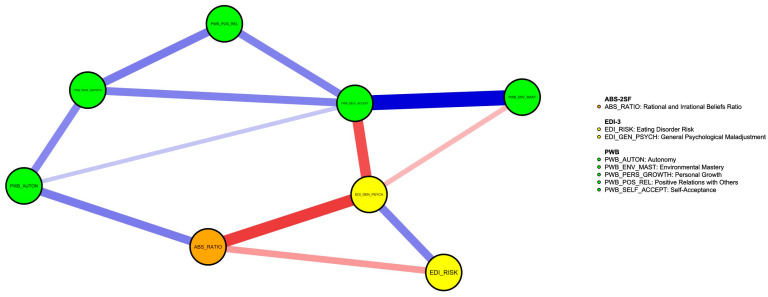
Network model of ED symptomatology, SOM ratio and psychological well-being. Blue lines represent positive correlations between nodes, while red lines represent negative correlations between nodes. Thicker lines represent stronger correlations.

**Figure 2:**
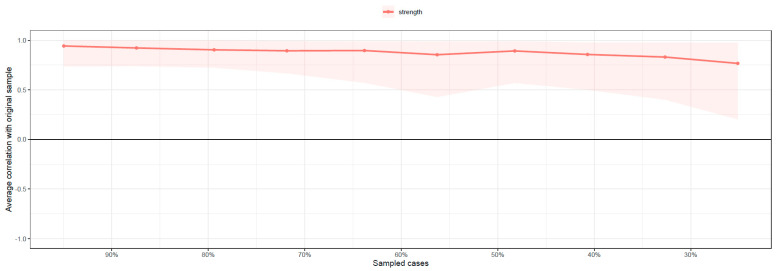
Bootstrapped strength centrality stability.

Controls for topological overlap of included nodes highlighted the significant shared proportion of edges between PWB personal growth and PWB purpose in life, leading to the exclusion of the latter from the network. Bootstrap operations for strength centrality and edge stability returned adequate results (CSC = 0.28; ESC = 0.67). Please see Figure 1 for a graphical representation of the network structure and Figure 2 for the bootstrapped strength centrality stability graphical representation.

**Figure 3:**
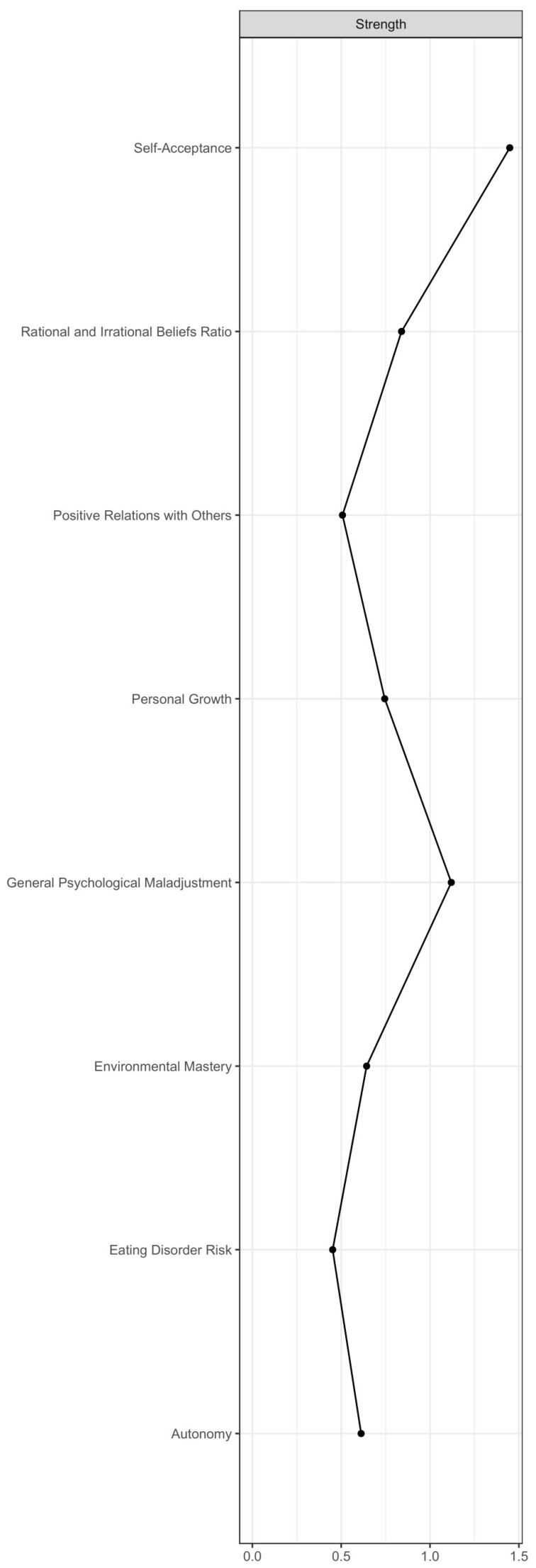
Complete strength centrality plot.

**Figure 4:**
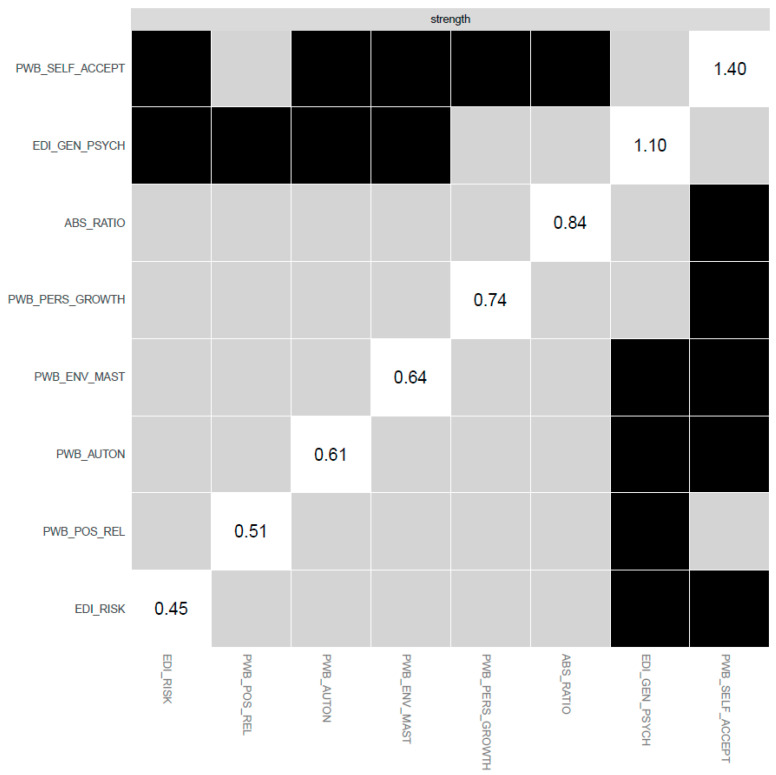
Strength centrality differences (significant differences in black).

The items with the highest strength centrality (i.e., items that were core to the SOM-EDI3-PWB network) were PWB self-acceptance, that is, one’s own self-satisfaction and awareness of strength and weaknesses or lack of it (PWB_SELF_ACCEPT; strength: 1.9), EDI-3 general psychological maladjustment (EDI_GEN_PSYCH; strength: 0.9), and SOM ratios of ABS-2 rational and ABS-2 irrational beliefs (ABS_RATIO; strength: 0.1). See Figure 3 for the strength centrality plot. The bootstrapped difference test, however, returned significant differences from other nodes only for PWB self-acceptance (PWB_SELF_ACCEPT) and EDI-3 general psychological maladjustment (EDI_GEN_PSYCH). See Figure 4 for the full table of significant differences between the network nodes’ strength values.

**Figure 5:**
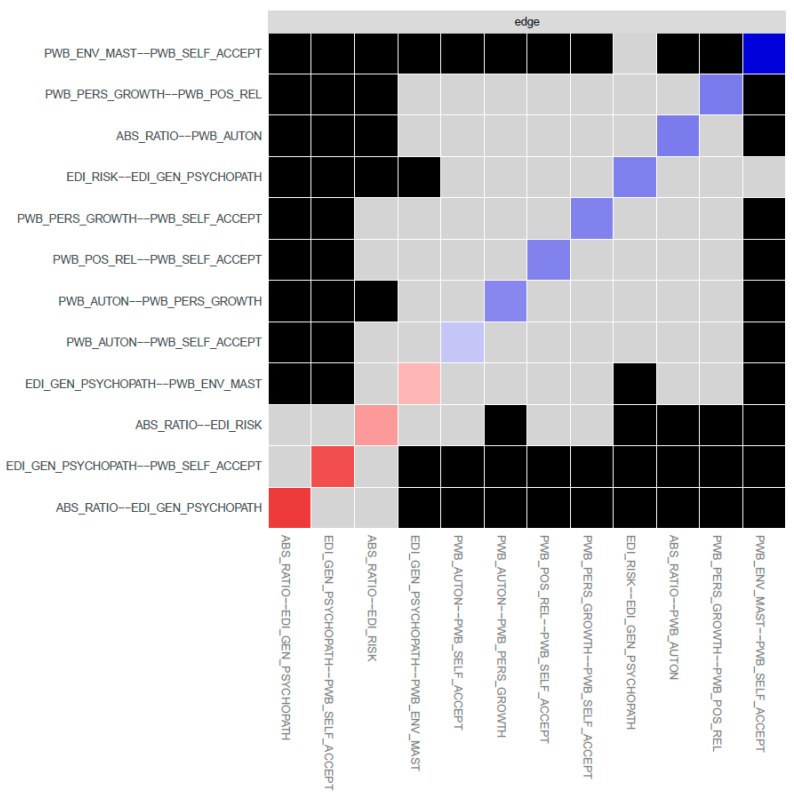
Bootstrapped edge weights differences (significant differences in black).

The individual edges with the highest weights in the network were the positive edge, connecting PWB self-acceptance (PWB_SELF_ACCEPT) and PWB environmental mastery (PWB_ENV_MAST) (weight: 0.49), and the negative edge, connecting SOM ratios of ABS-2 rational and ABS-2 irrational beliefs (ABS_RATIO) and EDI-3 general psychological maladjustment (EDI_GEN_PSYCH) (weight: −0.37). SOM ratios of ABS-2 rational and ABS-2 irrational beliefs (ABS_RATIO) connected with ED symptomatology (EDI_RISK and EDI_GEN_PSYCH) only through negative edges, while the connection with PWB autonomy (PWB_AUTON) was through a positive edge. See Table 3 for the complete report of all edge weights for the network. Bootstrap difference test returned significant differences for both edges with almost all other edges of the network. See Figure 5 for the full table of bootstrapped edge weight differences.

## 4. Discussion

The current study is the first to investigate the SOM model in EDs altogether and to conduct comparisons between a clinical ED sample and a control sample using the SOM model applied to cognitions. Moreover, it is the first to apply network analysis to examine the relationship between functional and dysfunctional cognitions, ED psychopathology, and psychological well-being in EDs.

The first hypothesis, according to which ED outpatients would exhibit less positively skewed beliefs (thus more compromised SOM cognitive balance), was supported. Indeed, ED patients exhibited lower scores in both rational beliefs and in the SOM ratio compared to controls, supporting the clinical utility of using the SOM ratio in the clinical assessment of cognitions in EDs and its ability to differentiate ED patients from the general population. The results are consistent with other works that applied the SOM model in non-clinical populations in which participants with emotional distress had lower SOM scores [32,33,34,35]. The current data are also in line with the ED literature, according to which ED patients present greater endorsement of irrational beliefs of catastrophizing, low frustration tolerance, and self-downing [16,60] and negative self and core beliefs [10,14], as well as a lower endorsement of positive core beliefs and positive attributes (Stein and [10,61] compared with healthy counterparts. In more recent work, ED-symptomatic individuals reported significantly greater endorsement of maladaptive core beliefs compared with the non-ED subgroup, in particular in self-loathing, unassertive, abandoned, and demanding subscales [18].

The second hypothesis concerned the centrality of SOM cognitive balance in the psychological network of EDs. We also hypothesized that the network would highlight a strong negative link between SOM cognitive balance and ED-related psychopathology. Both hypotheses have been supported by the NA data. The centrality of SOM cognitive balance, ED-related global psychological maladjustment and their significant association are generally in line with previous studies that found that worse SOM cognitive balance, that is, more negatively skewed thoughts, was significantly correlated with greater psychopathology, specifically in terms of worse anxiety and depression symptoms [29,31]. Similar constructs of maladaptive core beliefs have been found in the literature to contribute to increased vulnerability for developing eating psychopathology [62], in particular binge eating, purging, and dietary restriction [14,63], as well as predict disordered eating [64]. The current data suggest that more positively skewed cognitions could potentially protect against more severe ED-related psychopathology or inversely could reflect a psychopathological clinical picture characterized by a less severe ED profile. In line with the CBT and REBT theories [19,65,66] psychopathology is indeed theorized to be favored by both an inability to challenge and refute dysfunctional thoughts and an inability to formulate alternative functional thoughts, necessary for lasting cognitive change.

Furthermore, we hypothesized that the network would show a strong link between SOM cognitive balance and psychological well-being, specifically in terms of a significant positive relationship between SOM cognitive balance and psychological well-being. While a direct and significant link between SOM ratio and self-acceptance did not emerge, the network model suggests the importance of maintaining a positive attitude towards oneself and a positive general evaluation of one’s life (i.e., self-acceptance) to mitigate the impact of global psychological maladjustment related to ED and how the latter is strongly connected to worse cognitive balance. The network analysis results did however highlight the centrality and importance of self-acceptance in the definition of psychological well-being in EDs and its close link with ED-related psychopathology. Research suggests that low self-acceptance is closely associated with greater ED pathology and severity [24], in line with theoretical models that place a core negative self-belief at the basis of EDs, as the ED itself represents an attempt to achieve a false sense of self-worth [67]. Additionally, a substantial body of data on recovery criteria of ED patients underscores how patients themselves frequently consider self-acceptance and developing a positive sense of self as fundamental criteria for recovery from EDs, in addition to considering remission of eating pathology [68]. A lack of self-acceptance, according to the literature [24,68], would thus be at the basis of ED psychopathology and, inversely, a high degree of self-acceptance could buffer ED-related symptomatology.

Although not the strongest link in the network, the network results also suggested that greater SOM cognitive balance, that is, more positively skewed cognition, is positively associated with a greater sense of autonomy. A significant relationship had been reported in the general population between greater positively-skewed SOM ratios and positive functioning in terms of greater psychological adjustment and health [30,31]. Autonomy in the PWB model is understood as being self-determining, independent, able to resist social pressures, and able to think and act and regulates behavior from within [50]. Greater cognitive balance might reflect the individual’s ability to function independently of judgement from others by managing negative thinking more effectively, thus allowing a greater sense of autonomy to take hold. This finding is of clinical significance, as a lack of autonomy characterizes all ED patients and is associated with worse ED symptomatology [24] and is generally seen as a risk factor for ED [69,70], while gaining a sense of autonomy and independence from others’ judgements is cited by patients as both a motivator [71,72] and a fundamental criterion for recovery from the ED itself [68,73]. Moreover, the findings also suggest that bolstering a greater cognitive balance could in turn strengthen one’s self-acceptance or vice versa, indirectly activating other domains of psychological well-being, including environmental mastery, also central to ED recovery [25,68].

### 4.1. Implications

The use of the SOM model and ratio in assessing cognitive balance in ED has several advantages that have been previously posited. The consideration of both functional and dysfunctional cognitions, or more generally including a bidimensional assessment of cognition [29], allows one to capture the ability to self-regulate one’s thinking and internal dialogue through counteracting negative thinking with a functional counterpart. The SOM ratio thus can assess a more dynamic process of cognitive adaptation rather than focusing exclusively on the reduction of negative thinking, which would result in ignoring a crucial aspect of adaptive thinking.

The simultaneous examination in the SOM index of both types of cognitions (functional and dysfunctional) concurs with the concept of euthymia [74] and the dual continua model of mental health [75], whereby both positive functioning (i.e., psychological well-being) and psychological dysfunction and distress are not to be seen as mutually exclusive but rather as two independent dimensions that can co-exist. Consequently, the absence of ill-being does not necessarily coincide with the presence of wellness [74].

Furthermore, in clinical practice, the SOM might be used to instill realistic expectations in patients and clinicians as well, so that the goal in therapy becomes striving for healthy balance rather than having as a goal an absence of negative thinking, which is inevitable [29] and constitutes a limited measure of healthy and balanced thinking. This may be particularly important in those ED patients who might interpret persistence of negative cognitions as non-response to treatment, thus leading to low motivation or drop-out. Moreover, patients themselves refer that recovery from EDs should include not only improved cognitions but also acquiring the ability to manage emotions that distorted thinking elicit [68]. Finally, in ED patients, clinical practice may benefit from a broadening of assessment and case conceptualization that goes beyond the exclusive focus on ED symptomatology to include cognitive balance during pre- and post-treatment assessment, given its close associations with positive functioning, as underscored in the current study.

### 4.2. Limitations

The current study has several limitations. Firstly, it was not possible to consider diagnostic differences of ED subgroups due to power limitations; these might indeed present differential associations between the considered constructs. This might be the case for psychological well-being in particular, which varies across diagnostic groups [24], while irrational beliefs have not been found to differ between ED groups [16]. Longitudinal data would be needed to further support the utility of SOM ratio applied to rational and irrational beliefs in capturing worse ED profiles in terms of treatment outcomes. Additionally, results might have been magnified by the exclusion of controls with past ED diagnoses and the use of a convenience, not randomly selected, clinical sample. Moreover, the network strength coefficient reached only the adequacy threshold due to the limited sample size compared with the number of nodes included in the network. On other hand, the network analysis section of this study gains strength from the strict adherence to all five criteria for network studies’ methodological quality assessment and transparency proposed by Tomei and colleagues [76] based on the work of Burger and colleagues [58]. Future studies might benefit from the inclusion of male patients, as the numbers of males with EDs are on the rise [77], despite females being affected disproportionately [3].

## 5. Conclusions

Understanding the role of both adaptive and maladaptive cognitions in psychological functioning is an essential aspect of CBT practice. It offers further insights into patients’ cognitions, which may aid the much-needed improvement of CBT in treatment settings in this clinical population [78,79,80].

Future research might elucidate whether the SOM ratio is clinically useful in inpatient and hospital settings and investigate whether it longitudinally captures processes of cognitive change that occur from a negative interior dialogue to a positive interior dialogue during the course of psychotherapy [29,81], as seen in social phobia [36] and agoraphobia [37]. Indeed, “cognitive change” in the CBT research literature in general remains poorly defined.

## Figures and Tables

**Table 1:** Means of ED patients in psychological measures.

Measure	ED PATIENTS (n = 199) (M ± SD)
EDI-3 ED Risk (range 0–100)	52.69 ± 21.30
EDI-3 ED General Psychological Maladjustment (range 0–256)	98.50 ± 39.08
PWB Autonomy (range 14–84)	49.72 ±13.41
PWB Environmental Mastery (range 14–84)	46.30 ±13.04
PWB Personal Growth (range 14–84)	59.82 ± 11.56
PWB-Positive relations with others (range 14–84)	56.60 ± 13.33
PWB Life Purpose (range 14–84)	51.96 ± 13.67
PWB Self-Acceptance (range 14–84)	41.95 ± 14.50

Note: ED—eating disorder; EDI-3—eating disorder inventory-3; M—mean; PWB—psychological well-being scales; SD—standard deviation.

**Table 2:** *t*-test comparisons in SOM ratios and rational beliefs scores between ED patients and matched controls.

	ED PATIENTS (n = 199)	CONTROLS (n = 95)			
**Measure**	M ± SD	M ± SD	*t* (*df*)	*p*	*d*
**ABS-2 RATIONAL BELIEFS**	79.84 ± 25.13	99.81 ± 24.11	*t*(289) = 6.44	<0.0001	−0.80
**SOM RATIO**	0.54 ± 0.18	0.67 ± 0.17	*t*(241) = −6.36	<0.0001	−0.73

Note: ABS-2—attitudes and beliefs scale-2; *d*—Cohen’s d effect size; *df*—degrees of freedom; ED—eating disorder; M—mean; *p*—significance level; SD—standard deviation; SOM—states of mind; *t*—independent *t*-test.

**Table 3:** Individual edge weights table (strongest edges marked with *).

	ABS_RATIO	EDI_RISK	EDI_GEN_PSYCHOPATH	PWB_AUTON	PWB_ENV_MAST	PWB_PERS_GROWTH	PWB_POS_REL	PWB_SELF_ACCEPT
ABS_RATIO	0.00	−0.20	* −0.37	0.25	0.00	0.00	0.00	0.00
EDI_RISK	−0.20	0.00	0.25	0.00	0.00	0.00	0.00	0.00
EDI_GEN_PSYCHOPATH	* −0.37	0.25	0.00	0.00	−0.14	0.00	0.00	−0.34
PWB_AUTON	0.25	0.00	0.00	0.00	0.00	0.23	0.00	0.11
PWB_ENV_MAST	0.00	0.00	−0.14	0.00	0.00	0.00	0.00	* 0.49
PWB_PERS_GROWTH	0.00	0.00	0.00	0.23	0.00	0.00	0.26	0.24
PWB_POS_REL	0.00	0.00	0.00	0.00	0.00	0.26	0.00	0.24
PWB_SELF_ACCEPT	0.00	0.00	−0.34	0.11	*0.49	0.24	0.24	0.00

## Data Availability

Due to the nature of this research, participants of this study did not agree for their data to be shared publicly, so supporting data are not available.
